# Artificial intelligence-assisted organoid construction in congenital heart disease: current applications and future prospects

**DOI:** 10.3389/fbioe.2025.1691972

**Published:** 2025-12-10

**Authors:** Fang Chen, Hao-Yi Zhang, Yan-Long Wan, Jia-Nan Jia, Rui-Zhen Wang, Cheng Gao, Zhen-Yu Chao, Yu-Hua Ru, Zhe Wang, Kai Cheng, Jiong Zhang, Juan Feng, Jin-Ling Ren, Dong-Rui Ma, Zhen-Qiang Zhang

**Affiliations:** 1 Department of Medical School, Henan University of Chinese Medicine, Zhengzhou, China; 2 Academy of Chinese Medical Sciences, Henan University of Chinese Medicine, Zhengzhou, China; 3 National Clinical Research Center for Hematologic Diseases, Jiangsu Institute of Hematology, The First Affiliated Hospital of Soochow University, Suzhou, China; 4 Institute of Blood and Marrow Transplantation, Collaborative Innovation Center of Hematology, Soochow University, Suzhou, China; 5 Department of Radiology, Massachusetts General Hospital, Harvard Medical School, Boston, MA, United States

**Keywords:** congenital heart disease (CHD), cardiac organoids, artificial intelligence (AI), three-dimensional (3D) bioprinting, microfluidic technology

## Abstract

Congenital heart disease (CHD) is a complex group of cardiac abnormalities arising during fetal development. Despite advancements in diagnostics and surgery, CHD mechanisms remain elusive due to inadequate disease models. Recent innovations in artificial intelligence (AI)-assisted organoid construction, which replicate tissue architecture and function, provide a promising *in vitro* platform for modeling cardiac development and CHD progression with high precision. This review summarizes AI-driven approaches in CHD organoid construction, focusing on machine learning (ML) applications in self-assembly, three-dimensional (3D) bioprinting, tissue engineering, and microfluidic organ-on-a-chip (OOC) technologies. We also discuss refinements in AI algorithms - such as support vector machines (SVMs), decision trees, and neural networks - to enhance cell-cell interaction analysis, optimize drug screening, and improve toxicity/efficacy assessments. Looking ahead, AI is poised to accelerate CHD organoid translation to clinical practice, advancing precision medicine.

## 1 Introduction

Congenital heart disease (CHD) is the most common birth defect worldwide, affecting approximately 1% of newborns each year (Rodríguez-Pérez et al., 2025). Infants with CHD are at increased risk of developmental delays and a higher prevalence of related disorders (Rodríguez-Pérez et al., 2025), and some suffer from autonomic dysfunction (Govindan et al., 2025). Alarmingly, the 1-year survival rate for children with severe CHD is less than 80% (Oster et al., 2013). The etiology of CHD is multifactorial, involving genetic, environmental, and epigenetic factors. Recent advances in genomic technologies—such as third-generation sequencing and genome-wide association studies—have revealed that genetic abnormalities play a predominant role in CHD, particularly in cardiac development (Tafazoli et al., 2025).

However, CHD research remains limited by the lack of effective experimental models that accurately recapitulate genetic abnormalities and mimic the complex developmental processes of the human heart (Rodríguez-Pérez et al., 2025; Oster et al., 2013). Although animal models share many developmental similarities with humans, they differ significantly in gene expression patterns and overall cardiac structure (Cao et al., 2025). *In vitro* cell models can simulate certain aspects of heart development but fail to reproduce the *in vivo* cellular microenvironment or the intricate physiological functions of human organs (Li J. et al., 2022).

In recent decades, developmental and stem cell biology have advanced considerably, deepening our understanding of the molecular mechanisms governing stem and progenitor cell behavior. At the same time, progress in regenerative medicine has demonstrated the potential of stem cells to repair damaged tissues, paving the way for the reconstruction of functional organs in vitro—structures known as *organoids*. Benefiting from insights into extracellular matrix (ECM) biology, the development of three-dimensional (3D) culture systems, and innovations in suspension culture techniques, organoids have evolved from simple cell aggregates into complex 3D structures featuring vascular networks, neural innervation, and immune microenvironments (Yaqinuddin et al., 2025). Despite successful generation of organoids for various organs - including the heart, kidney, brain, and skin - the establishment of cardiac organoids for CHD remains challenging due to the structural complexity of the heart and the difficulties associated with subsequent data analysis (Rossi et al., 2018).

Artificial intelligence (AI), a pivotal branch of computer science, aims to develop computational systems capable of performing tasks that typically require human intelligence (Hamet and Tremblay, 2017). Machine learning (ML), a core subset of AI, has been widely applied across diverse fields. In biomedical research, ML algorithms can enhance image segmentation and analysis, classify and segment medical images, assist in constructing 3D organ models, optimize biomaterial selection, and predict cell behavior and tissue durability.

Although AI applications in medicine have expanded rapidly, systematic reviews focusing on AI-driven CHD research remain limited. One major challenge is the high degree of inter-individual variability in CHD organoids, whereas most AI algorithms rely on large volumes of homogeneous data, which are currently scarce. Nevertheless, AI technologies are reshaping the landscape of CHD diagnosis and treatment, and their integration with organoid research offers a transformative approach to studying this complex disease.

This review provides a comprehensive overview of cardiac organoid technology, emphasizing the potential synergies between AI and organoid models in CHD research. We highlight current protocol inconsistencies, propose strategies for AI integration, and identify future directions to bridge experimental innovation with clinical application, thereby encouraging further exploration in this emerging field.

## 2 Disease organoid models in CHD

CHD encompasses a broad spectrum of structural cardiac anomalies, including atrial and ventricular septal defects, abnormal heart valve development, and patent ductus arteriosus. Due to the heart’s complex architecture, morphology, and diversity of tissue types, the progress in constructing cardiac organoid models has been relatively slow, lagging behind advancements achieved in other organs such as the brain and kidney (Qian et al., 2019; Takasato et al., 2015). Nevertheless, recent research employing cardiac organoids has successfully established robust models that recapitulate specific CHD subtypes, including atrioventricular septal defects, valvular malformations, and related disorders (Lewis-Israeli et al., 2021; Khademhosseini et al., 2007; Wolfe et al., 2023; Miyamoto et al., 2021).

### 2.1 Cell sources for CHD organoid modeling

The human heart is composed of multiple specialized cell types that work in coordination to maintain normal cardiac function. In developing CHD organoid models *in vitro*, researchers aim to select cell sources capable of supporting balanced cardiomyocyte (CM) formation and angiogenesis, thereby producing viable and functional tissue structures. The ultimate goal is to construct human cardiac organoids with comparable structural and cellular complexity to the native heart (Salih et al., 2024).

Theoretically, pluripotent stem cells (PSCs), including embryonic stem cells (ESCs) and induced pluripotent stem cells (iPSCs), can be used to generate CHD organoids. PSC-derived cardiomyocytes have already been employed in studies of cardiac development, hereditary heart diseases, and therapeutic screening (Rao et al., 2022). Embryonic stem cells, isolated from human blastocysts, represent another key source for heart organoid generation. ESC-derived cardiac organoids can differentiate into multiple cardiac cell types and have been instrumental in elucidating cellular defects underlying CHD, suggesting that ESCs may serve as an excellent resource for CHD modeling (Rao et al., 2022). However, ethical constraints and concerns regarding immune rejection have limited the practical application of ESCs, making iPSCs a more feasible alternative for research and potential clinical use.

Induced pluripotent stem cells (iPSCs), reprogrammed from adult somatic cells, can differentiate into cardiomyocytes, endothelial cells (both blood and lymphatic), cardiac fibroblasts, and vascular smooth muscle cells (Salih et al., 2024). Notably, iPSC-derived valvular endothelial and stromal cells exhibit high expression of the transcription factor Gata4, which induces endothelial-to-mesenchymal transition. This process leads to the formation of the endocardial cushion in the outflow tract and promotes the development of cardiac semilunar valves (Koenig et al., 2016). Such findings demonstrate that iPSCs can effectively recapitulate key developmental processes involved in valvular malformations and septal defects - pathologies that constitute a significant proportion of CHD cases. Consequently, a range of iPSC-derived models for atrioventricular septal defects and valve malformations have already been developed using first-generation autologous cells (Cao et al., 2025; Rao et al., 2022).

Furthermore, iPSC-derived cardiac organoids can be constructed *in vitro* by mimicking essential cardiac parameters, including chamber formation, balanced organization of cardiomyocytes and vascular networks, and electrophysiological properties (Lee et al., 2022). These models can partially reproduce the electrophysiological abnormalities characteristic of CHD, providing a valuable platform for investigating disease mechanisms and potential therapeutic interventions.

### 2.2 Methods for constructing cardiac organoid models

Currently, four main technological systems are used to construct organoids: self-assembly, 3D bioprinting, tissue engineering, and microfluidic organ-on-chip platforms. Each approach offers distinct advantages and, together, they have driven substantial progress in organoid development. An overview of these methods is provided below.

#### 2.2.1 Self-assembled cardiac organoids

Self-assembled organoids are organ-like structures formed through the spontaneous aggregation, differentiation, and organization of cells with minimal external manipulation (Lewis-Israeli et al., 2021). In the context of cardiac organoid generation, the Wnt (Wingless) signaling pathway - a critical regulator of embryonic development and tissue morphogenesis - has proven particularly valuable. This pathway typically operates through three sequential regulatory phases (activation–inhibition–activation) and plays an essential role in directing pluripotent stem cell (PSC) differentiation toward cardiac lineages.

During organoid formation, modulation of Wnt signaling is often achieved by sequential exposure to CHIR99021, a Wnt activator that inhibits GSK3, and Wnt-C59, a Wnt inhibitor that blocks PORCN-mediated signaling. When applied under optimal timing and concentration conditions, this combination promotes efficient cardiogenic mesoderm induction from induced pluripotent stem cells (iPSCs). The resulting cardiac organoids display physiologically relevant ratios of epicardial and myocardial tissues, closely mimicking the complexity of the human heart (Lewis-Israeli et al., 2021). Lewis-Israeli et al. developed a protocol using Matrigel-based suspension cultures to generate PSC-derived, self-organizing early embryonic human heart organoids. Their method involved three distinct stages of Wnt modulation at specific developmental time points (Lewis-Israeli et al., 2021; Lee et al., 2022; Volmert et al., 2023). The resulting organoids contained epicardial, endocardial, endothelial, fibroblast, and cardiomyocyte (CM) populations - with approximately 59% of the cells identified as CMs - and exhibited gene expression profiles highly similar to those of human fetal hearts (Lewis-Israeli et al., 2021).

Similarly, Hofbauer et al. successfully generated heart-shaped, chamber-like organoids from PSCs containing one to three cardiac cell lineages. These simplified structures, devoid of non-cardiac tissue interference, offer a tractable model for studying early human cardiac morphogenesis and disease pathogenesis (Hofbauer et al., 2021). In another study, (Volmert et al., 2023) applied multiple induction strategies - ranging from standard maturation media to enhanced maturation media (1 and 2/1) - to early embryonic cardiac organoids. Organoids cultured in enhanced maturation medium 2/1 showed the closest correlation to human cardiac tissue, reflecting improved maturation and functional development.

The self-assembly approach enables large-scale and high-throughput generation of cardiac organoids, providing statistically robust data that can be leveraged for drug screening and investigation of genetic or teratogenic mechanisms underlying CHD. However, this method still faces significant limitations: the cellular composition, tissue architecture, and degree of maturation of the resulting organoids cannot yet be precisely controlled, posing challenges for reproducibility and physiological fidelity.

#### 2.2.2 3D bioprinting in cardiac organoids

3D bioprinting involves four key steps: bioink formulation, model design, printing, and functional regulation. As an emerging technology, 3D bioprinting offers the ability to create customized structures based on patient-specific imaging data, allowing for the production of anatomically accurate models. Once implanted, these structures have the potential to grow and remodel, making them particularly promising for applications in CHD (Salih et al., 2024).

The use of computer-aided design and computer-aided manufacturing technologies allows for the precise, layer-by-layer construction of biomimetic 3D structures derived from medical images. This method ensures the accurate deposition of multiple cell types, biomaterials, and biomolecules, which can be tailored to construct cardiac organoid models. The controlled deposition of these components into tissue structures offers the potential for building complex, functional models of the human heart.

Among various 3D bioprinting techniques, scaffold-free bioprinting is particularly notable. This method involves the deposition of cell spheroids onto a needle array to construct tissues without requiring extracellular matrix (ECM)-based materials. The primary advantage of scaffold-free bioprinting is its high speed, enabling rapid tissue fabrication (Salih et al., 2024). For instance, researchers have successfully utilized scaffold-free 3D bioprinting to create tubular engineered heart tissues using human-induced pluripotent stem cells (hiPSCs). These tissue constructs were capable of spontaneous beating and could be electrically paced after transplantation into rats. This innovation helps overcome issues associated with traditional artificial grafts, such as infection susceptibility and thrombosis (Kawai et al., 2021).

Cardiac organoids generated via 3D bioprinting can incorporate perfusable vascular networks, making them particularly relevant for regenerative medicine applications, where tissue viability and function rely on blood vessel formation (Fang et al., 2023). However, despite its potential, the cost of 3D bioprinting remains high, and achieving large-scale printing is still a significant challenge. Additionally, cell loss during the printing process is common, which can limit the functionality of the printed tissue. Thus, while 3D bioprinting holds tremendous promise, further optimization of the technology is necessary to enhance reproducibility and scalability (Wang et al., 2024a).

#### 2.2.3 Tissue engineering in cardiac organoids

Tissue engineering approaches for constructing cardiac organoid models involve the use of biomaterials, scaffolds, extracellular matrices (ECMs), and bioengineering devices to culture and inoculate a variety of cell types derived from stem cells. This methodology not only aids in generating organoid models but also provides a platform for pathophysiological testing (Yang et al., 2023). Successful tissue engineering requires careful consideration of seed cells, supporting cell scaffolds, and bioactive factors that regulate cellular behavior (Yang et al., 2023).

Early attempts at cardiac tissue engineering focused on removing non-myocytes from primary cardiac cells (Bursac et al., 1999). However, subsequent studies have demonstrated that varying the inoculation ratios of cardiomyocytes (CMs), cardiac fibroblasts, and cardiac endothelial cells (ECs) in cardiac organoids can improve model fidelity. Researchers compared two methods: pre-culture (where ECs and fibroblasts were cultured for 2 days in microchannels before CM inoculation) and simultaneous triple-culture (where all three cell types were seeded simultaneously). This comparison revealed that incorporating a specific proportion of non-myocytes during the pre-culture phase enhanced the structure and function of the resulting cardiac organoids (Iyer et al., 2009).

Though tissue-engineered 3D cardiac models typically involve fewer cell types and more complex construction processes, they have shown great promise in analyzing cardiac contractility, electrophysiology, and other critical aspects (Yang et al., 2023). While these models are not yet ideal for disease modeling, they provide a valuable platform for investigating the pathogenesis of CHD.

#### 2.2.4 Microfluidic technology in cardiac organoids

Microfluidic technology involves creating biomimetic microfluidic systems designed to precisely control fluid flow and nutrient supply at the micron scale, simulating the physiological environment *in vivo* (Saorin et al., 2023). When combined with organoid culture, microfluidics facilitates the development of organ-on-a-chip (OOC) systems, enabling the modeling of organs in both healthy and pathological states with high controllability (Saorin et al., 2023).

Recent advances in microfluidic technology include the development of heart-kidney devices, where human iPSC-derived heart and kidney organoids are cultivated in a one-way flow and closed perfusion system. This setup allows for the creation of multi-organ *in vitro* models (Gabbin et al., 2023). In one study, human iPSC-derived heart and kidney organoids were loaded into two independent chambers within the perfusion chip. Results showed that organoids cultured under dynamic perfusion conditions retained their structural and functional properties compared to those in static culture, highlighting the advantages of microfluidic systems for organoid development (Gabbin et al., 2023).

However, a current limitation of microfluidic-based organoid models is the lack of vascularization, which restricts the depth of disease modeling, especially for conditions requiring blood vessel formation. Despite this challenge, microfluidic systems are expected to play a crucial role in the study of organ-organ interactions *in vitro* (Zhao et al., 2019). They offer a novel approach to studying physiological and pathological mechanisms that are difficult to replicate in traditional tissue systems, and could provide valuable insights into disease pathogenesis and potential therapeutic strategies, particularly for diseases involving multiple organ systems (Gabbin et al., 2023).

**FIGURE 1:**
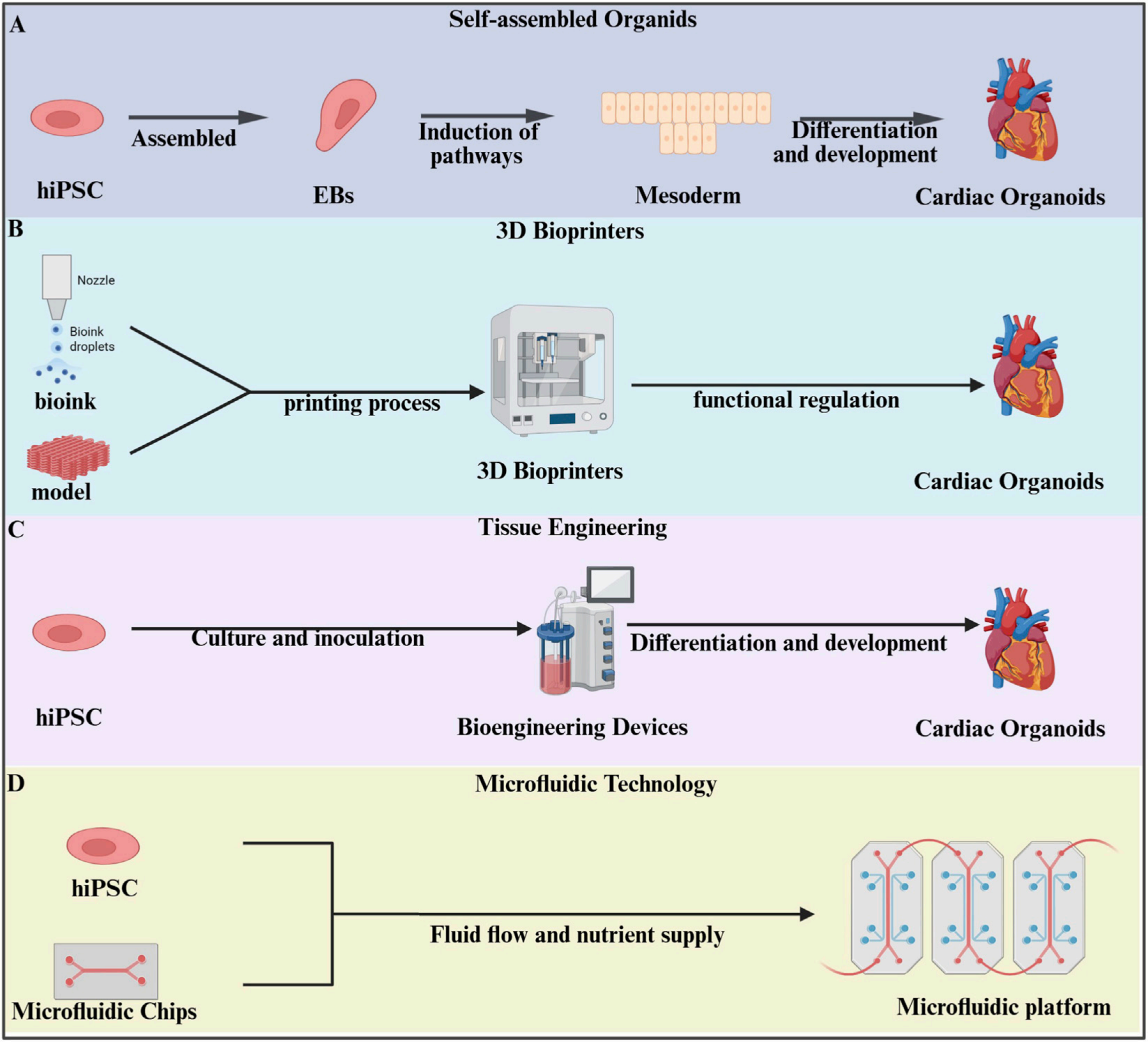
Methods of constructing organoids. **(A)** Human induced pluripotent stem cells (hiPSCs) were directed to form suspension embryoid bodies for mesoderm differentiation into cardiac organoids (Lewis-Israeli et al., 2021; Lee et al., 2022; Volmert et al., 2023). **(B)** Three-dimensional(3D) bioprinting technology utilizes specialized bioprinters to process prepared bioinks according to pre-designed digital models, enabling the fabrication of cardiac organoids (Kawai et al., 2021). **(C)** HiPSCs were cultured and inoculated into bioengineering devices for further induction of cardiac organoids (Yang et al., 2023). **(D)** Microfluidic technology integrates organoids into microfluidic chips, establishing a precisely controlled platform through fluid dynamics and nutrient delivery, ultimately enabling casual self-organization of cultured cells into functional organoids on chip (Saorin et al., 2023).

Here, we summarized the detailed methods for constructing cardiac organoids in Figure 1 and Table 1.

**TABLE 1:** Summary of existing cardiac organoids.

Architecture	Materials	Cell composition	Modulated pathway	Applications	Ref.
Self-organizing early embryonic human heart organoid	Matrigel	CMs CFs ECs Epicardial cells Endocardial cells	Wnt	Pharmacological screenings	Lewis-Israeli et al. (2021)
Multi-chamber cardioid	CDM	CMs ECs	Wnt Nodal/Activin BMP	Dissecting genetic and teratogenic causes of human cardiac defects	Volmert et al. (2023)
Scaffold-free tubular engineered heart tissue	MEM-α, endothelial basal medium-2, fibroblast growth basal medium-2	CMs HUVECs NHDFs	None	Myocardial regeneration therapy	Hofbauer et al. (2021)
Vascularized heart tissue	Matrigel	CMs ECs FBs	None	Tracking cardiac tricultures of cell	Kawai et al. (2021)
Cardiac and kidney organoids	E8 medium	CMs CFs ECs	None	Disease modeling	Yang et al. (2023)

CMs, cardiomyocytes; CFs, cardiac fibroblasts; ECs, endothelial cells; HUVECs, human umbilical vein endothelial cells; NHDFs, normal human dermal fibroblasts; FBs, fibroblasts; CDM, chemical defined medium; MEM-α, 5% fetal bovine serum/minimum essential medium-α.

## 3 AI-assisted cardiac organoid construction in CHD

Artificial Intelligence (AI) has become a transformative technology, simulating human intelligence through advanced computational systems. It enables machines to perform tasks traditionally requiring human cognitive functions such as reasoning, learning, problem-solving, and language comprehension (Wang et al., 2023). In healthcare, AI’s ability to analyze vast datasets, identify complex patterns, and generate actionable insights has revolutionized medical practice, enhancing diagnostic accuracy, operational efficiency, and therapeutic decision-making (Flynn and Chang, 2024; Owoyele et al., 2025). A prime example of AI’s impact is its application in medical imaging, where AI-driven systems achieve remarkable precision in disease detection, enabling earlier interventions and improving patient outcomes (Miyoshi, 2025). In the context of CHD, machine learning (ML) - a core component of AI - has proven invaluable in predictive modeling and optimizing processes like cardiac organoid development (Wang et al., 2024b).

### 3.1 Common ML models in the medical field

Machine learning models provide essential support in solving complex problems across various domains, including healthcare, through their advanced computational and mathematical approaches (Bai et al., 2024). Among the many types of ML models, three commonly used ones in the medical field are support vector machines (SVM), decision trees, and neural networks.

Support Vector Machine (SVM): SVMs are pivotal in medical imaging and molecular biology, where they are used for feature extraction and classification. They work by creating optimal decision boundaries (hyperplanes) in high-dimensional spaces, making them particularly useful for tasks like text classification and bioinformatics, where the number of features is much higher than the number of observations. For example, J. Sharma et al. applied SVM to predict heart failure with 79% prediction accuracy (Sharma et al., 2024). SVM’s strength lies in its ability to handle high-dimensional data, making it ideal for complex healthcare applications (Balfer et al., 2014; Yao and Huang, 2022).

Decision Trees: Decision trees are supervised learning algorithms commonly used for classification and regression tasks (Ebrahim and Derbew, 2023; Pashaei et al., 2015). They function by recursively splitting data into subsets based on input feature values, resulting in a tree-like structure of decisions. Each node in the tree represents a feature, each branch represents a decision rule, and each leaf node represents an outcome. Decision trees are valued for their simplicity and interpretability, making them highly useful in applications where understanding the decision-making process is critical, such as in medical diagnoses and financial predictions (Mambetsariev et al., 2018; Rooij et al., 2015).

**FIGURE 2:**
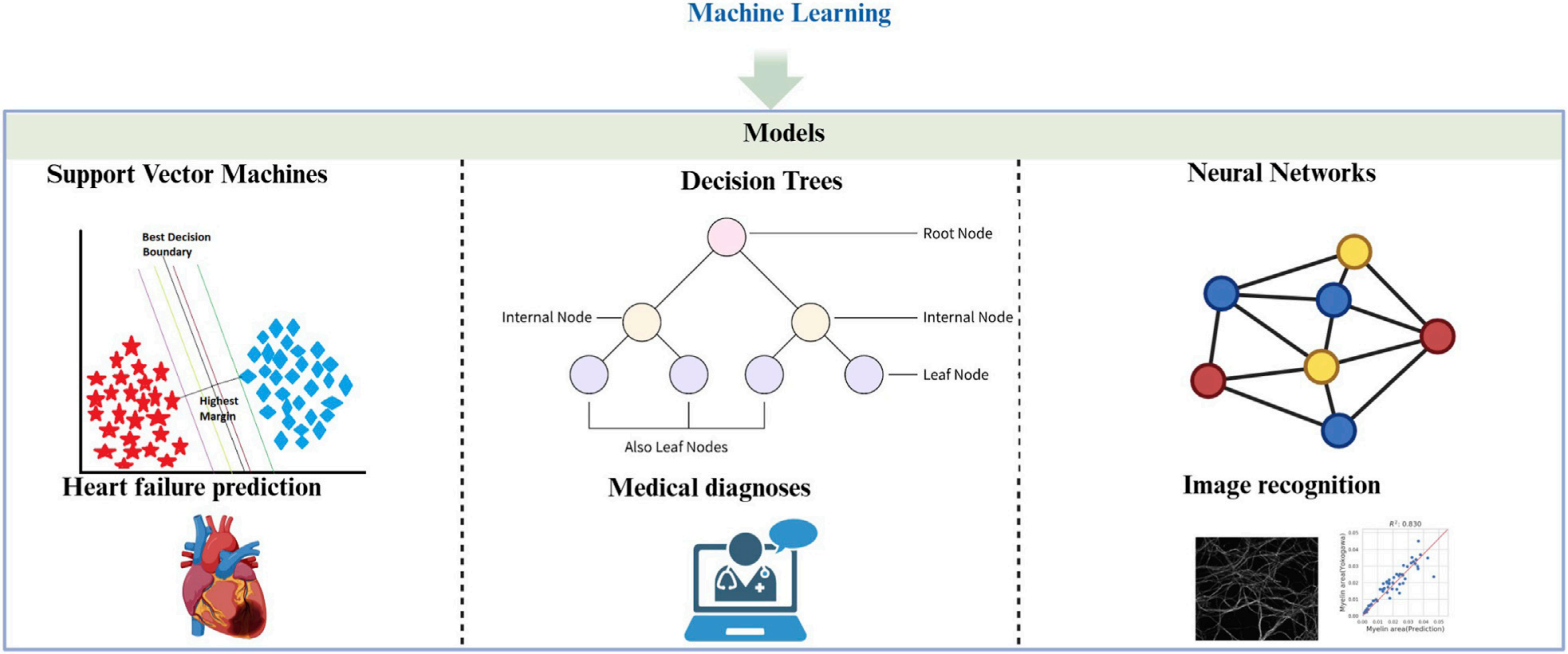
Machine learning (ML). A description of the common ML models and their applications in medical field. Support Vector Machines (SVMs) techniques have shown significant applications in the field of heart failure prediction (Chen et al., 2024). Decision Trees can be suitable for medical diagnosis (Mambetsariev et al., 2018; Rooij et al., 2015). Neural Networks can be applied to histopathology image recognition (Xiong et al., 2021).

Neural Networks: Neural networks, particularly Convolutional Neural Networks (CNNs), have shown unprecedented potential in medical image analysis (Li H. et al., 2022). CNNs are highly effective in detecting disease-related patterns in histopathology images, learning hierarchical spatial features to accurately identify conditions such as cancer (Wang and Hu, 2022). Recurrent neural networks, on the other hand, excel at processing sequential data, and have been applied in medical report generation and text prediction in electronic health records (Esteva et al., 2019; Ren et al., 2022). Additionally, graph neural networks have gained traction in biomedical research for capturing complex relationships in biological networks, aiding in the prediction of intermolecular interactions and drug development (Xiong et al., 2021) (Figure 2).

### 3.2 Application of AI/ML in CHD organoid construction

AI technology is playing an increasingly powerful role in CHD research, particularly in the development of CHD organoid models. By integrating AI/ML tools, the process of constructing these organoids has become more efficient, accurate, and scalable. Specifically, AI can significantly enhance various aspects of organoid development, from modeling and material selection to process optimization and functional evaluation.

3D Bioprinting of Cardiac Organoids: In 3D bioprinting, AI helps in creating precise 3D models of cardiac structures, guiding the optimal selection of bioinks, and fine-tuning printing parameters in real-time. These interventions can vastly improve the morphological fidelity and structural integrity of the resulting cardiac organoids. By ensuring the accurate reproduction of anatomical features, AI-assisted 3D printing allows for more reliable and reproducible models of CHD (Chen et al., 2024; Arnaout et al., 2021; Chen et al., 2023; Zhang et al., 2022; Jin et al., 2023).

Tissue Engineering for Cardiac Organoids: In tissue engineering, machine learning (ML) plays a crucial role in predicting the differentiation purity of cardiomyocytes (CMs) based on limited or historical data. ML algorithms can also be used to quantitatively assess the morphology and contractile function of differentiated cardiomyocytes. Additionally, AI can evaluate the durability of engineered cardiac valves, which are vital for studying the functional aspects of congenital valve defects and other CHD-related conditions (Guo et al., 2023; Williams et al., 2020; Pasqualini et al., 2015; Lee et al., 2015; Liang and Sun, 2019).

Microfluidic Technology in Cardiac Organoids: AI/ML applications in microfluidic technology are diverse. These include the intelligent design of chip structures, dynamic regulation of cell culture conditions, and the efficient processing of complex data from experiments. One of the most impactful contributions of AI in microfluidics has been its role in fluorescence microscopy image analysis, which now benefits from enhanced automation and accuracy. These advancements improve the precision and reproducibility of results in organoid research (Stoecklein et al., 2017; Sabaté Del Río et al., 2023; Li J. et al., 2022; Zhou et al., 2020; Cascarano et al., 2021).

While the self-assembly approach for organoid construction is not discussed here due to limited literature, the other three methods - 3D bioprinting, tissue engineering, and microfluidic technology - have seen significant AI contributions.

In Table 2, we summarize the technical directions, core algorithms, and practical outcomes of AI applications in different CHD organoid construction methods.

**TABLE 2:** Summary of AI/ML applications in different construction methods of CHD organoids.

Construction method	AI application direction	Specific ML technology/Algorithm	Application scenario/Effect	Ref.
Self-assembly	With limited applications and few relevant literature, this method will not be discussed	——	——	——
3D Bioprinting	1. 3D structure modeling 2. Bioink selection 3. Printing process optimization 4. Quality control	1. DL, intelligent segmentation, image enhancement tools 2. Decision trees, random forests, DL 3. Six-degree-of-freedom robotic arm and layered growth strategy 4. Convolutional neural networks with image processing technology	1. Constructing high-precision cardiac structure models based on magnetic resonance imaging and computed tomography scans images, with 95% sensitivity in CHD screening 2. Predicting biomaterial printability with 88% accuracy, optimizing ratios of collagen, gelatinetc. 3. Improved vascularization of cardiac organs, addressing deep tissue nutrient supply issues 4. Real-time monitoring of transparent hydrogel defects and automatic adjustment of printing parameters	Chen et al (2023), Arnaout et al (2021), Chen et al (2024), Zhang et al (2022), Jin et al (2023)
Tissue Engineering	1. Cardiomyocyte culture 2. Myocardial morphology assessment 3. Cardiomyocyte contraction assessment 4. Evaluation of tissue Engineered flaps	1. Random forest, Gaussian process regression algorithms 2. Bayesian classifiers, feed-forward neural networks, and bootstrap aggregation algorithms 3. Optical flow algorithm, SVM 4. ML	1. Predicting PSCs cardiac differentiation purity with 84% accuracy using only 16% of early-stage differentiation data 2. Providing more reliable algorithms for cell maturity assessment 3. Extracting 12 contraction parameters, achieving 83%–99% accuracy in drug toxicity classification under high concentration conditions 4. Replacing accelerated wear tests with accuracy comparable to finite element methods and improved computational efficiency	Guo et al (2023),Williams et al (2020), Pasqualini et al (2015), Lee et al (2015), Liang and Sun (2015)
Microfluidic organ-on-chip	1. Chip design and culture condition optimization 2. Data analysis 3. Image detection	1. Computer vision integrated with machine learning-based electrochemical detection 2. DL 3. Recursive deep prior video algorithm	1. Optimizing chip structure and material selection, dynamically adjusting medium components to extend organ function maintenance time 2. Simplifying data analysis processes and addressing challenges posed by a large amount of data 3. Solve the delayed microscope resolution in OOC applications	Stoecklein et al. (2017), Sabaté Del Río et al. (2023), Li J.et al. (2022), Zhou et al. (2020), Cascarano et al. (2021)

DL, deep learning; CHD, congenital heart disease; SVM, support vector machine; ML, machine learning; PSCs, pluripotent stem cells; OOC, organ-on-a-chip.

#### 3.2.1 AI in the construction of self-assembly CHD organoids

The Wnt-BMP signaling axis in the mesoderm plays a crucial role in regulating cardiac cavity morphogenesis during the formation of cardiac organoids. Studies have shown that induced pluripotent stem cells (iPSCs) can form cardiac organoids without the need for external scaffolds, relying on self-assembly mechanisms (Kaushik et al., 2019). While AI’s role in this area remains relatively limited, one potential future direction could involve using AI to predict biological behavior through the modulation of the Wnt-BMP signaling axis. This would help optimize organoid formation by simulating the complex signaling events driving heart development, thus enhancing the self-assembly process.

#### 3.2.2 AI-enhanced construction of CHD organoids using 3D bioprinting

3D bioprinting has emerged as a powerful technology for constructing heart-like organoids, leveraging its ability to combine cells and biomaterials through layer-by-layer deposition. This approach has significant potential for bio-fabrication, but it faces several challenges, including maintaining structural integrity, ensuring cell viability and functionality, and improving reproducibility (Chen et al., 2024). AI can play a pivotal role in overcoming these challenges by optimizing various aspects of 3D bioprinting. Specifically, AI can: Optimize cardiac organ models based on medical imaging. Predict and optimize bioink composition, ensuring the material’s compatibility with cellular growth. Monitor and adjust printing parameters in real-time to ensure high-quality, reproducible prints (Chen et al., 2024; Erps et al., 2021; Yu and Jiang, 2020). These capabilities enable the production of more reliable and anatomically accurate cardiac organoids, advancing the field of CHD research.

##### 3.2.2.1 AI-assisted 3D structural modeling of cardiac organoids

Accurate 3D modeling of cardiac organoids is critical, especially when patient-specific data is used to design the organoid structures. The primary source of such data comes from MRI and CT scans, which need to be processed precisely for optimal modeling. A critical aspect of this is medical image classification and segmentation, which identifies and isolates diseased tissue, helping to replicate the pathological state in the organoid model (Greil et al., 2007; Sodian et al., 2007).

For instance, Rima Arnaout et al. trained a deep learning (DL) neural network system using retrospective echocardiography and fetal screening ultrasound data to detect fetal CHD. This system achieved impressive diagnostic results, including a sensitivity of 95% (95% confidence interval 84%–99%) and a specificity of 96% (95% confidence interval 95%–97%), with a negative predictive value of 100% (Ma et al., 2023). Compared to traditional methods, this DL-based model demonstrated higher sensitivity and improved data processing efficiency, thus enhancing diagnostic accuracy.

**FIGURE 3:**
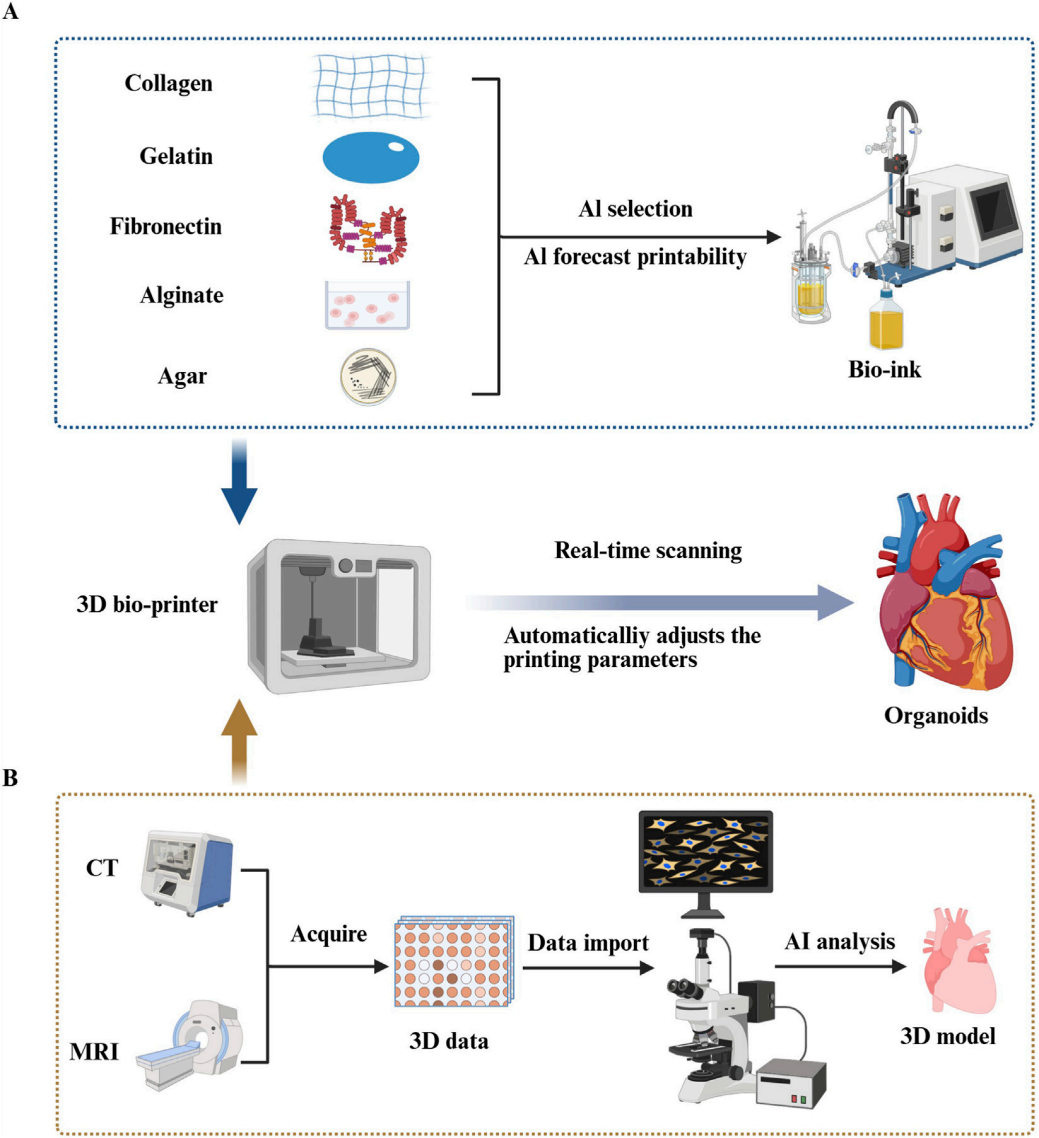
Artificial intelligence (AI) improves congenital heart disease (CHD) organoid construction in three-dimensional (3D) bioprinting. **(A)** Using various machine learning (ML) algorithms including decision trees, random forests, and deep learning (DL), researchers can accurately predict the printability of bioinks formed by different proportions of biomaterial combinations (Chen et al., 2023; Ma et al., 2023; Jafari et al., 2022). (Such as collagen, gelatin, fibronectin, alginate, agar.) **(B)** AI can precisely segment medical images obtained from Magnetic Resonance Imaging and Computed Tomography scans, thereby extracting 3D data of target tissues and constructing high-precision 3D models of target organs through computer-aided design systems (Greil et al., 2007; Sodian et al., 2007). By integrating real-time imaging systems into bioprinting devices and combining ML technology to analyze and automatically evaluate the collected image data in real time, the system can intelligently adjust printing parameters based on feedback, ensuring printing quality and precision (Goh et al., 2021; Bonatti et al., 2024).

The integration of this technology not only aids in cardiac view recognition and coronary heart disease screening, but also significantly enhances the construction of pathologically relevant cardiac organoid models. By accurately reflecting disease states, AI-powered image analysis helps create organoids that more closely resemble human CHD pathology (Figure 3).

##### 3.2.2.2 AI-assisted bioink selection

The selection of bioink materials is critical for bio-3D printing, as the material choice directly impacts the structural integrity, cell viability, and functionality of the printed tissue. A variety of natural and synthetic materials are used in bioinks, including collagen, gelatin, fibronectin, alginate, agar, and hydrogels, each offering unique advantages (Jafari et al., 2022). Developing the optimal bioink formulation often requires iterative experimentation and optimization, considering complex parameters such as gelling behavior, degradation rate, and biocompatibility (Elbadawi et al., 2024; Nadernezhad and Groll, 2022).

Machine learning (ML) techniques have emerged as powerful tools to streamline this development process. In one study, Chen et al. used ML models, including decision trees, random forests, and deep learning (DL), to predict the printability of 210 ink formulations, comprising 16 different bioactive materials and 4 solvents. The study found that deep learning (DL) achieved the highest recall rate (87.3%), outperforming other models in terms of accuracy and reliability (Chen et al., 2023). This demonstrates the potential of AI to accurately evaluate the printability of biomaterials, enabling researchers to select the most suitable bioinks for the 3D printing process of cardiac organoids. In doing so, AI can significantly enhance the precision and efficiency of bioink selection, which is critical for advancing cardiac tissue engineering (Figure 3).

##### 3.2.2.3 AI-enhanced 3D bioprinting process

###### 3.2.2.3.1 AI-driven improvements in bioprinting technology

Traditional 3D bioprinting faces significant challenges that hinder its practical applications, particularly when it comes to spatial flexibility during printing and maintaining cell viability and functionality post-printing. These limitations have constrained the broader development of bioprinting technologies. However, the integration of AI provides innovative solutions to these issues.

For example, Zhang et al. developed an advanced bioprinting system that combines a six-degree-of-freedom robotic arm with a bio-3D printing platform to address issues of vascularization and sustained cell survival during organoid manufacturing. The system incorporated an innovative “layered growth” strategy, which allowed cells to grow and form functional connections between print intervals. This method facilitated extracellular matrix secretion and promoted the formation of blood vessels within the printed tissue.

The biomimetic printing approach not only enhanced vascularization but also ensured that the tissues remained alive and continued to beat for over 6 months. This development significantly improved the deep tissue feeding challenge and has opened up new avenues for advancing tissue engineering and cardiac organoid research (Zhang et al., 2022). Such breakthroughs illustrate how AI-driven bioprinting systems can enhance the spatial complexity and long-term viability of cardiac tissues, bringing us closer to functional *in vitro* organ models (Figure 3).

###### 3.2.2.3.2 AI for quality control and on-site monitoring in bioprinting

Bio-3D printing technology relies heavily on precise control of parameters such as printing speed, temperature, and material extrusion rate (Goh et al., 2021). Traditional methods of manual parameter regulation often fall short due to the complexity and variability of these factors. To address these challenges, one effective solution is the integration of real-time imaging systems with machine learning (ML) algorithms for continuous quality monitoring during the printing process (Bonatti et al., 2024; Hsieh and Hsu, 2019).

The key advantage of this approach is its ability to continuously optimize algorithmic systems by processing a large volume of sample data. As data accumulates, the system’s detection sensitivity and accuracy improve, providing real-time, intelligent feedback to guide the bioprinting process. For example, Jin et al. developed a deep learning (DL) detection system that combines CNNs with image processing techniques to monitor the quality of printed structures. The system analyzes real-time images captured during printing to detect and classify various printing defects, such as issues with structure, density, and morphology in transparent hydrogel-based bioinks.

This DL-based system can accurately identify the location and type of defects in printed structures and offers the potential to automatically adjust printing parameters based on the inspection results. In their study, Jin et al. reported an overall accuracy of 90.1% and an F1-score of 0.955 when testing the system on a validation dataset (Jin et al., 2023). These results suggest that the integration of real-time defect detection with AI-based adjustments could significantly improve the quality and precision of bioprinting, ultimately enhancing the reliability and reproducibility of cardiac organoid models (Figure 3).

#### 3.2.3 AI in the construction of CHD organoids in tissue engineering

The concept of tissue engineering, first proposed in 1980, centers around creating biological substitutes for tissues or organs that can be used in clinical applications (Berthiaume et al., 2011). As a multidisciplinary field, tissue engineering involves three core elements: seed cells, support matrix, and growth factors. Recent advancements in tissue engineering, particularly in cardiac tissue engineering, have shown great promise in improving the clinical prognosis of cardiovascular diseases, including congenital heart defects (Bagherpour et al., 2025; Langer and Vacanti, 2016).

Machine learning (ML) has been successfully applied across multiple areas of tissue engineering, including the development of biomaterials, the fabrication of scaffolds, and tissue biology (Guo et al., 2023). Among various applications, ML has been most intensely used in the engineering of prosthetic heart valves, where it addresses several technical challenges: cell culture optimization, myocardial morphology assessment, contraction evaluation, and predicting the persistence of valve types (Kusumoto and Yuasa, 2019; Rafieyan et al., 2023). Below are specific examples of how AI can enhance different aspects of cardiac tissue engineering in CHD organoid construction.

##### 3.2.3.1 AI assisting cardiomyocyte culture

###### 3.2.3.1.1 AI for optimizing cardiomyocyte culture conditions

Purification through metabolic selection is a widely used method to obtain highly pure cardiomyocytes (CMs) with the lipid metabolism characteristics typical of adult heart cells (Sharma et al., 2015). However, while this method has proven effective in many cases, it does not guarantee the success of the differentiation process and is often resource-intensive and time-consuming.

The process of cardiac differentiation is complex and not fully understood, leading to challenges in predicting outcomes and optimizing methods. ML techniques offer a solution by enabling the identification of key factors that influence differentiation and predicting outcomes with higher accuracy.

For example, Williams et al. applied ML to model data from the human pluripotent stem cell-derived CM differentiation process in a stirred tank bioreactor. The model analyzed variables such as dissolved oxygen, pH, and cell density from 58 experiments. With this approach, the model could predict by the 5th day whether the cardiomyocyte production on the 10th day would be “adequate,” achieving a prediction accuracy of 85%. By the 7th day, the accuracy improved to 90% (Williams et al., 2020).

This ML-driven approach not only provides deeper insights into the cardiac differentiation process but also significantly reduces costs, improves the predictability of outcomes, and makes differentiation results more measurable and reproducible.

###### 3.2.3.1.2 AI-assisted assessment of myocardial morphology

Morphological studies have shown that the structure of cardiomyocytes (CMs) is closely linked to their function. Mature CMs typically have an elongated, rod-like shape with aspect ratios ranging from 7:1 to 9.5:1. In contrast, hiPSC-CMs (human induced pluripotent stem cell-derived CMs) often display a rounded or polygonal morphology, with significantly lower aspect ratios (Gerdes et al., 1992). Traditional methods of assessing cardiac morphology can be labor-intensive, time-consuming, and prone to subjective bias. To address these issues, machine learning (ML)-based image analysis techniques have greatly improved the efficiency and objectivity of CM morphology assessment (Kalkunte et al., 2024).

For example, Pasquilini et al. assessed cell maturation by staining for α-actinin and fibronectin. Eleven key morphological metrics were evaluated, including module length, stacking density, and Z-disk consistency. The researchers employed Bayesian classifiers, feed-forward neural networks, and bootstrap aggregation algorithms to process these 11 morphological metrics. The goal was to differentiate between mature and immature states of primary rat CMs. The three models achieved accuracies of 70%, 71%, and 77%, with high confidence, providing a more reliable approach to cell maturity assessment (Pasqualini et al., 2015).

These ML-driven approaches enable a more quantitative, consistent, and automated evaluation of CM morphology, reducing reliance on subjective interpretation.

###### 3.2.3.1.3 AI-assisted cardiomyocyte contraction assessment

Mature CMs feature well-established calcium regulation mechanisms mediated by voltage-gated ion channels for calcium, sodium, and potassium. These channels regulate intracellular calcium release and reuptake, enabling efficient contraction-relaxation cycles (Bodi et al., 2005). HiPSC-CMs, however, exhibit calcium cycling defects, leading to weaker contractile function (Kalkunte et al., 2024). This difference is evident when measuring contractile stress: adult ventricular CMs generate more than 50 mN/mm^2^, neonatal CMs produce 0.8–1.7 mN/mm^2^, while hiPSC-CMs only generate 0.15–0.30 mN/mm^2^.

The maturation of myocardial contractile function is regulated by a complex network of factors. Lee’s team was among the first to apply ML to analyze cardiomyocyte function in 2015. They identified cell behavior using sequences of bright-field images via SVMs. The study employed an optical flow algorithm to extract cell motion vectors, which were then simplified using principal component analysis into a single dynamic variable. The team extracted 12 contraction parameters, including peak duration, amplitude, and frequency, and used these parameters to train the SVM model to differentiate between normal and abnormal cell states.

In tests involving drugs like E-4031, verapamil, and blebbistatin, the SVM model achieved classification accuracy ranging from 83% to 99% under high drug concentrations. However, its predictive ability dropped significantly when the drug concentration was below 10 nM (Lee et al., 2015). Despite this limitation at low drug concentrations, the model’s performance at higher concentrations remains noteworthy and holds significant potential for drug screening and cardiac function analysis.

##### 3.2.3.2 AI-assisted evaluation of tissue-engineered flaps

Tissue-engineered heart valves are classified as FDA Class III medical devices, and their development typically involves long timelines and high costs. Traditional methods for evaluating the durability of these devices rely on time-consuming accelerated wear tests and expensive animal studies (Liang and Sun, 2019; Martin and Sun, 2015). These traditional approaches are not efficient enough to meet the demands of mass production, creating a critical need for more efficient, scalable evaluation systems.

Machine learning (ML) offers a promising solution by providing the ability to efficiently analyze biological stress processes, thereby accelerating the development of evaluation systems. One approach that has gained traction in this area is finite element analysis, which is widely used in the mechanical evaluation of autologous and biological valves to calculate stress distribution and deformation (Sacks et al., 2009; Sacks and Yoganathan, 2007).

In this context, Li’s team leveraged ML techniques to predict the stress and deformation of transcatheter aortic valve (TAV) leaflets based on their design parameters. The study constructed and compared two deep neural network models: one based on an autoencoder structure and the other a direct prediction model. These models were evaluated using Monte Carlo cross-validation, a robust technique for validating the models’ generalizability and predictive power.

**FIGURE 4:**
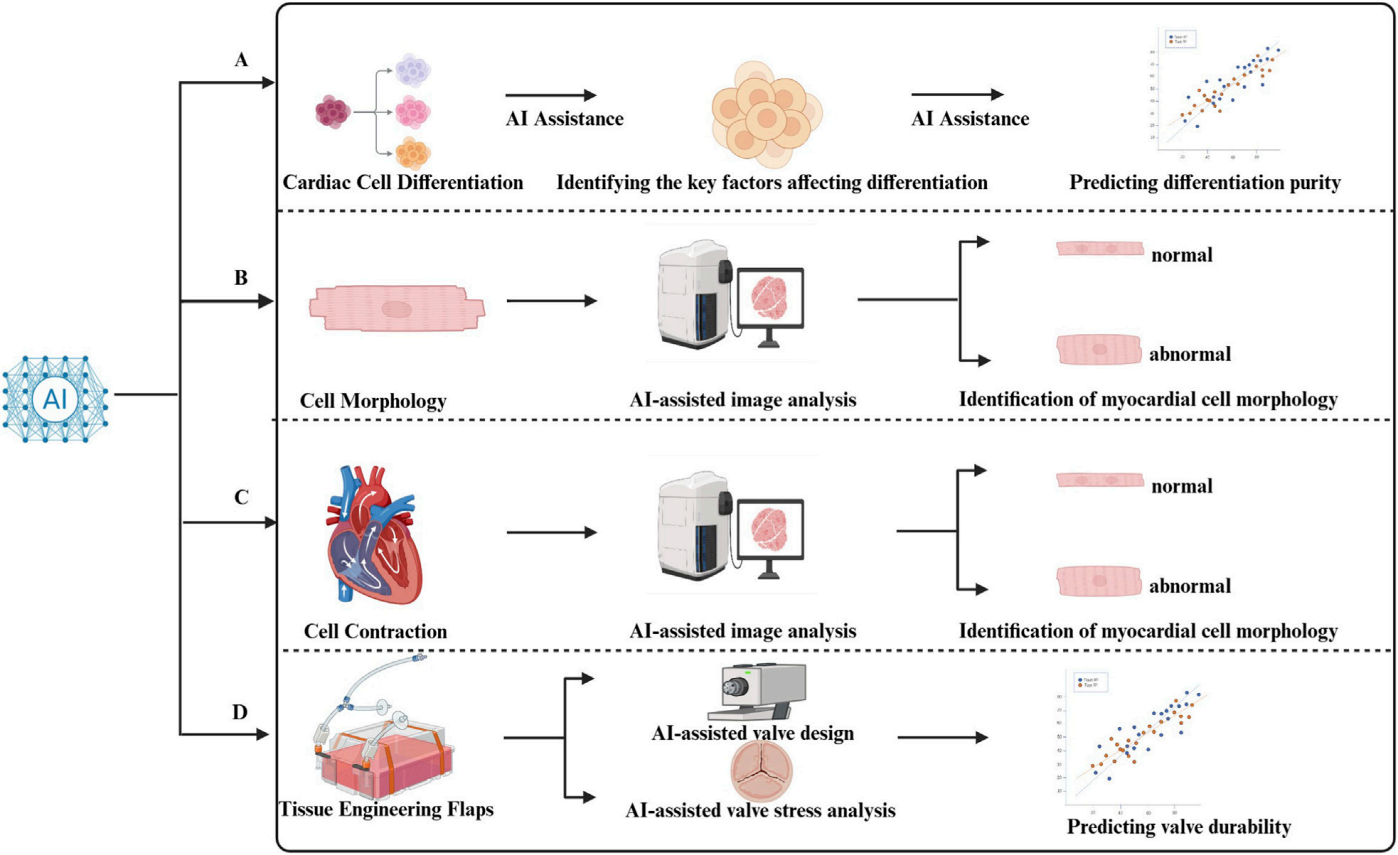
Artificial intelligence (AI) improved congenital heart disease (CHD) Tissue Engineering. **(A)** Machine learning(ML) can identify and record the key factors (such as dissolved oxygen concentration, cell concentration, power of Hydrogen value, etc.) in the differentiation process of cardiomyocytes (CMs). Through algorithms such as random forest and Gaussian Process Regression, complex biological processes can be transformed into computable mathematical models, so as to achieve accurate prediction of the purity of myocardial differentiation (Williams et al., 2020; Huang et al., 2024). **(B)** In cellular morphological analysis, AI can utilize morphological features of CMs (such as module length, module stacking density, and Z-disk consistency.) collected through specific staining methods, combined with Bayesian classifiers, feedforward neural networks, and guided aggregation algorithms, to conduct comprehensive assessment of cell maturity (Pasqualini et al., 2015). **(C)** By capturing high-definition images of CMs contractions and extracting key contraction parameters (Such as peak duration, amplitude, and frequency and so on), SVMs can effectively analyze these parameters to precisely distinguish between normal and abnormal CMs (Lee et al., 2015). **(D)** Specially trained ML can perform in-depth analysis of the deformation geometry and biomechanical properties of heart valves, providing important support for valve design optimization and durability assessment (Liang and Sun, 2019).

The results demonstrated that both ML models could accurately predict the deformation geometry and the corresponding stress distribution of TAV leaflets. While this work is still in the proof-of-concept stage, it clearly highlights the significant potential of machine learning in advancing the development of tissue-engineered valves by providing a more efficient, cost-effective, and scalable approach to valve evaluation (Figure 4).

#### 3.2.4 AI-enhanced construction of CHD organoids on microfluidic organ chips

Among the many breakthrough applications of microfluidic technology, organ-on-a-chip (OOC) systems stand out for their ability to replicate the microstructures and physiological environments of human organs. By combining micro-nanofabrication techniques with fluid dynamics, OOCs can simulate human organ function with high precision. These systems also integrate multi-sensing capabilities to monitor and regulate mechanical stresses, physiological signals, and chemical gradients, offering a powerful platform for biomedical research (Huang et al., 2024; Nithin et al., 2023).

With the rapid advancement of AI technology, particularly in visual recognition and data analysis, the field of microfluidics has gained new momentum. AI’s role in optimizing OOC design, cultivation conditions, and data handling has significantly advanced the potential of microfluidic organ models. The incorporation of AI has made it possible to optimize the design process, improve image tracking and detection, and manage large experimental datasets, thus pushing the innovation and development of microfluidic systems.

##### 3.2.4.1 AI-driven optimization of OOC design and culture conditions

In traditional microfluidic chip design, researchers often rely heavily on experience and trial-and-error. While this approach can yield results, it is time-consuming and can be limited by existing design paradigms. The introduction of AI, especially deep learning (DL) algorithms, has revolutionized this process. By leveraging AI, the design and material selection for organ-on-a-chip (OOC) systems can be optimized, making devices more customized for specific applications (Stoecklein et al., 2017). For example, AI-powered computer vision techniques, such as CNNs, have enabled rapid and accurate chip design, which significantly reduces errors during fabrication.

Optimizing culture media is another key challenge in the development of OOCs. The correct culture medium is essential for maintaining organ function, a problem that is particularly pronounced in multi-organ systems, where different cell types require distinct environments for optimal survival and function (Jalili-Firoozinezhad et al., 2021).

One significant issue faced by long-term cultures is the decline of organ function due to limited oxygen diffusion and the necrosis of tissue. To address this, researchers are developing vascularized models with perfusable vascular networks, which could lay the foundation for creating long-lasting, functional organoids (Deng et al., 2023; Galan et al., 2020). In this context, AI-driven systems have proven crucial. Specifically, electrochemical detection, a non-invasive method, has been used for real-time monitoring of parameters like nutrient levels, metabolites, growth factors, and pH levels in OOCs (Sabaté Del Río et al., 2023). By continuously collecting this data, AI can dynamically adjust culture media and conditions, significantly extending the functional lifespan of the organ models.

Moreover, AI isn’t limited to chemical parameters. In one study, Oliver J. Dressler’s team used deep Q-networks (a form of reinforcement learning) to analyze image feedback and automatically adjust the flow conditions in a microfluidic system. This capability is especially valuable in long-term experiments, where inconsistencies in system performance can arise. By automatically regulating the physical environment of culture fluids, this method offers a highly precise control strategy for OOC platforms (Dressler et al., 2018).

In summary, AI’s role in microfluidics is expanding far beyond post-experiment analysis. Today, AI is integral in both the design and real-time optimization of OOC systems, including the dynamic regulation of both chemical and physical parameters. This shift represents a significant transformation in the field, offering intelligent regulation and control of experimental environments, with profound potential for the future of organoid research.

##### 3.2.4.2 AI-enhanced OOC data throughput

###### 3.2.4.2.1 AI simplifies data analysis

As the field of organ-on-a-chip (OOC) technology advances and high-throughput, parallelized microfluidic systems become more widespread, the volume of data generated has grown exponentially. This data explosion has far outpaced researchers’ ability to process and analyze it using traditional methods, creating a significant bottleneck that hinders research progress (Riordon et al., 2019). Traditional data analysis techniques are often inefficient, error-prone, and can be influenced by subjective biases, which may lead to the omission of critical insights.

In this context, AI, particularly the application of deep learning (DL), has revolutionized data analysis. AI methods help automate and simplify the process of analyzing large, complex datasets, enabling researchers to extract valuable insights from experimental data that would otherwise be challenging to process (Li J. et al., 2022; Zhou et al., 2020). This advancement not only addresses the limitations of traditional methods but also opens up new possibilities for research by making it possible to tackle analysis tasks that were previously considered impractical. As a result, AI has become a vital tool for unlocking the full potential of OOC systems and improving the efficiency and accuracy of data analysis in biomedical research.

###### 3.2.4.2.2 AI improves image detection and segmentation

Fluorescence microscopy images are commonly used for data output in OOC experiments, providing detailed visual information about cellular interactions and other dynamic processes. However, analyzing these images manually has traditionally been time-consuming, labor-intensive, and highly prone to errors (Riordon et al., 2019). This is especially true for image segmentation, which is essential for quantifying and interpreting experimental results, such as identifying regions of interest, assessing cell morphology, or tracking drug effects.

To overcome these limitations, researchers have turned to deep learning techniques for automated image analysis. AI-based image segmentation algorithms offer several key benefits, including faster processing times, improved accuracy, and greater reliability, particularly in complex or large datasets. One promising development in this area is the Recursive Deep Prior Video (RDPV) algorithm, developed by Pasquale Cascarano et al. This innovative algorithm extends the well-known deep image prior technique to time-lapse microscopy, allowing for super-resolution imaging without requiring pre-trained models. The RDPV algorithm is specifically designed to address issues with microscope resolution in OOC experiments, enabling clearer, more detailed images for analysis (Cascarano et al., 2021).

**FIGURE 5:**
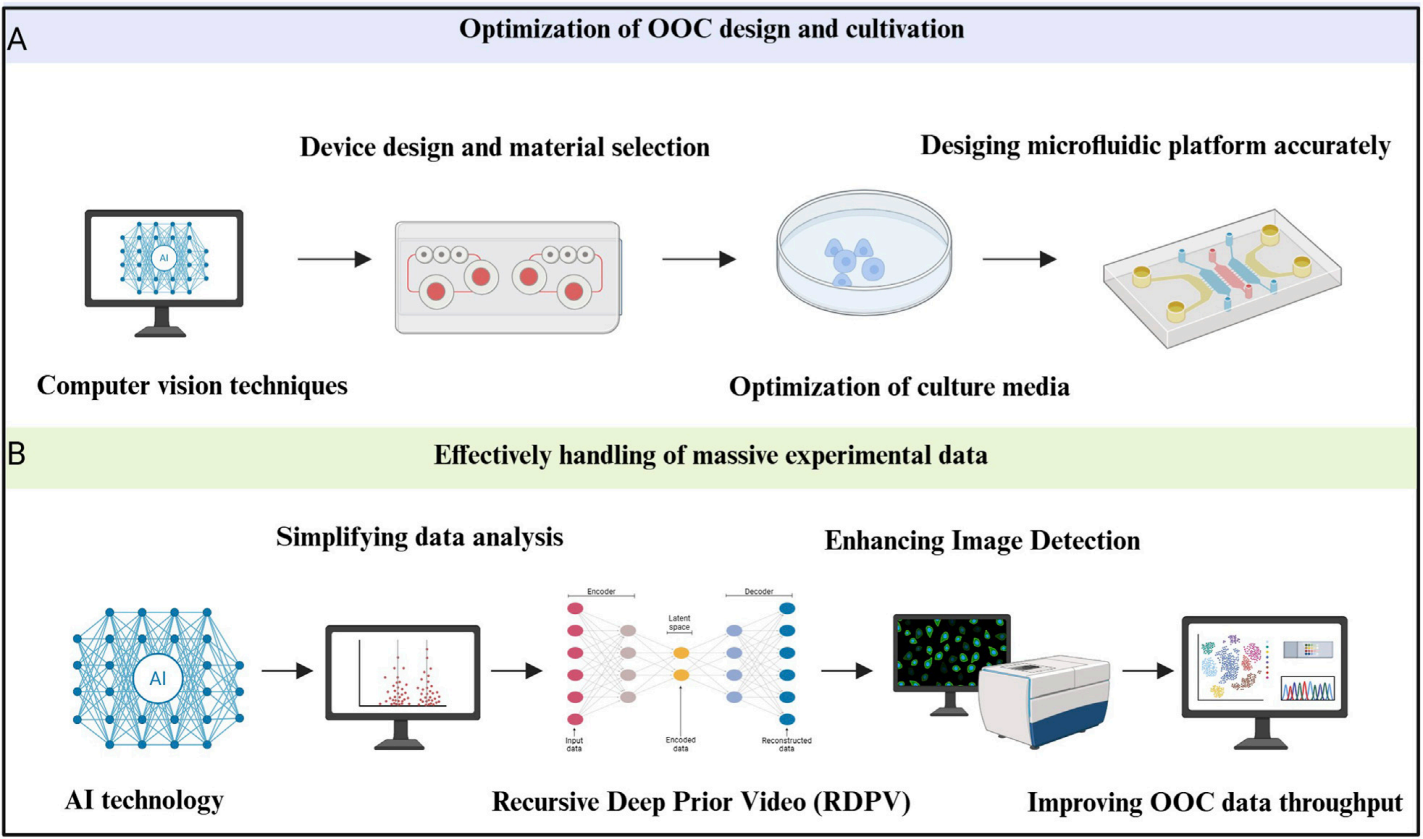
Artificial intelligence (AI) improves the construction of congenital heart disease (CHD) organoids in microfluidic organ chips. **(A)** Optimization of design and culture conditions with the help of AI. Applying computer vision techniques can make microfluidic device design and material selection quick and accurate. Meanwhile, AI can optimize culture media and then help design microfluidic platform accurately (Stoecklein et al., 2017; Sabaté Del Río et al., 2023). **(B)** Effectively handling massive experimental data with the help of AI means that deep learning (DL) greatly simplifies the data analysis process. Recursive Deep Prior Video can improve the efficiency of recognition and enhancing image detection to improve organ-on-a-chip (OOC) data throughput (Li J. et al., 2022; Zhou et al., 2020; Deng et al., 2023; Du and Dua, 2010).

This approach has been practically tested in OOC studies, such as those investigating tumor-immune interactions, where it demonstrated excellent performance and promising application potential in improving the quality and accuracy of fluorescence microscopy image analysis (Figure 5).

## 4 Discussion and future perspective

AI has the potential to greatly enhance both the efficiency of organoid construction and the accuracy of CHD model predictions by integrating various advanced technologies such as gene editing, image analysis, and multi-omics sequencing (Wang H. et al., 2024). Human cardiac organoid models, which incorporate multiple cell types into structured 3D assemblies, provide a powerful tool for mimicking *in vivo* organ function (Iyer et al., 2009). These models address the limitations of traditional 2D culture systems and the interspecies differences often encountered in animal models (Iyer et al., 2009). AI can further augment this progress by integrating multi-omics data (including genetic and epigenetic information) with organoid phenotypic data. This integration can help efficiently identify pathogenic variants and enable the identification of critical links between gene mutations and structural abnormalities in organoids, ultimately improving the accuracy of variant classification and aiding early risk prediction for CHD.

### 4.1 Drug discovery and screening

The synergy between AI and organoid technology holds immense promise for accelerating drug discovery, screening, and toxicity testing. By utilizing AI, researchers can significantly enhance drug efficacy and safety evaluations (Yao et al., 2020). Compared to animal-based cardiac models, self-assembled cardiac organoids offer several advantages, including higher throughput, reduced reliance on animal testing, and fewer ethical concerns, making them highly attractive platforms for preclinical drug testing (Bursac et al., 1999). Furthermore, AI can assist in identifying key signaling pathways critical for heart development, enabling researchers to streamline the fabrication of cardiac organoids, thus reducing both time and cost (Stiefbold et al., 2024).

### 4.2 Personalized medicine

The integration of AI with organoid technology has far-reaching implications for personalized medicine. CHD organoid models can be designed to represent specific cell populations, enabling more targeted studies of the pathophysiological states affecting distinct regions of the developing heart (Stiefbold et al., 2024). This approach supports dynamic, evidence-based clinical decision-making, transforming how individualized treatment strategies are developed. Notably, using cardiac organoids derived from induced pluripotent stem cells (iPSCs), AI can predict individual patient responses to drugs by analyzing electrophysiological and contractile data post-treatment (Lee et al., 2015). This has the potential to reduce clinical trial failure rates and could be expanded to research for rare forms of CHD, thus shortening the drug development cycles for specialized patient populations (Yao et al., 2020). Additionally, the integration of AI with microfluidic chips can enable real-time monitoring of organoid states, allowing for dynamic adjustments of drug concentrations based on experimental data (Sabaté Del Río et al., 2023). This capability could be extended to long-term post-operative efficacy tracking in patients, providing essential technical support for the ongoing optimization of personalized treatment regimens.

### 4.3 Challenges and future directions

Despite these advances, several challenges remain. One of the biggest hurdles is the biological complexity involved in developing accurate cardiac organoid models. Heart development is governed by intricate spatial and temporal interactions between genes, proteins, and various cell types (Haick and Tang, 2021; Bazzicalupi et al., 1996). Replicating these complex processes *in vitro* remains a significant challenge, particularly when constructing models that closely mimic cardiac morphogenesis (Bazzicalupi et al., 1996; Gu et al., 2022).

Another major issue is that the abundance of data does not guarantee its **usefulness**. Data from sources like smartphones or wearable devices may lack **scientific rigor**, leading to **biases** (Cahan et al., 2019). While **machine learning (ML)** excels at interpreting existing data, the ability to **predict long-term medical outcomes** is still fraught with uncertainty (Zhang et al., 2023). Furthermore, the **high operational costs** and complex requirements of organoid systems often contribute to **low inter-batch reproducibility**, which hampers **standardization** (Zhang et al., 2023).

Ethical concerns also play a crucial role. While organoid technology itself presents minimal ethical risks, the use of **patient-derived genetic data** requires careful handling to ensure **privacy** and **informed consent** (Du and Xie, 2021). Moreover, **biases** in AI training datasets—such as those related to **patient self-selection**, **confounding variables**, and **inconsistent clinical outcomes -** could lead to **demographic disparities** in predictions (Yao et al., 2020). For example, predictive models developed using predominantly **European** data, like those from the **Framingham Heart Study**, may not generalize well to other populations (Cahan et al., 2019). Therefore, it is critical to establish **ethical frameworks** that address **genetic privacy**, **data ownership**, and **consent** to ensure the responsible application of AI in this field.

### 4.4 Key challenges for the future

To fully unlock the potential of **AI-organoid integration**, several key challenges must be addressed, including:

**Data Standardization**: Ensuring data consistency across platforms and studies.

Interdisciplinary Collaboration: Encouraging cooperation between AI specialists, biologists, and engineers.

Computational Limitations: Overcoming the computational burden of processing large datasets.

Algorithm Interpretability: Ensuring AI models are transparent and their decision-making processes can be understood.

Ethical Governance: Establishing strong guidelines for the use of patient data and ensuring fairness in model training.

To tackle these issues, researchers must **curate high-quality training datasets**, **harmonize evaluation metrics**, and conduct **robust experimental validation** (Mara et al., 2024). Additionally, advances in **organoid engineering -** such as the use of **human-derived matrix materials**, the development of **OOC systems** to simulate **inter-organ communication**, and the application of **3D bioprinting** to overcome scaling limitations—will further enhance the **physiological relevance** of these models (Wang H. et al., 2024; Mara et al., 2024; Kusters et al., 2020).

Looking ahead, future AI research in this field should focus on: Transparency in model interpretability; Innovation in evaluation methodologies; The development of regulatory frameworks for AI applications in biomedical research (Kusters et al., 2020).

Emerging AI techniques, such as **fine-tuned pre-training methods**, which have shown success across various domains, hold great promise for organoid research. By leveraging knowledge from **related domains**, pre-trained models could significantly improve the **classification** of data in the target domain, even with **limited data**. This approach has the potential to outperform training models from scratch, reducing **data scarcity** issues and cutting **costs** associated with organoid-based investigations in CHD research.

## 5 Conclusion

In conclusion, while there are still challenges to overcome, AI technology holds immense potential to improve the **fidelity** and **scalability** of organoid-based research. Its integration into studies of **CHD** marks a significant milestone in advancing **clinical translation** and fostering the growth of **precision medicine** (Wu et al., 2024). By enabling more accurate predictions, personalized treatments, and accelerated drug development, AI is poised to play a transformative role not only in **CHD organoid modeling** but also in various areas of biomedical research. As interdisciplinary collaboration continues to evolve, the convergence of AI with organoid technologies will undoubtedly pave the way for groundbreaking innovations and clinical breakthroughs in the near future.

## Abbreviations

3D, three-dimensional; AI, artificial intelligence; CHD, congenital heart disease; CM, cardiomyocyte; CNNs, convolutional neural networks; DL, deep learning; ECM, extracellular matrix; ECs, endothelial cells; ESCs, embryonic stem cells; HIPSCs, human induced pluripotent stem cells; IPSCs, induced pluripotent stem cells; ML, machine learning; OOC, organ-on-a-chip; PSCs, pluripotent stem cells; RDPV, recursive deep prior video; SVM, support vector machine; TAV, transcatheter aortic valve.
